# Gaining community entry with survivors for forensic human rights and humanitarian intervention

**DOI:** 10.1080/20961790.2021.2002524

**Published:** 2022-02-10

**Authors:** Jaymelee Kim, Lucia Elgerud, Hugh Tuller

**Affiliations:** a Justice Sciences, University of Findlay, Findlay, OH, USA; b Anthropology, University of Tennessee-Knoxville, Knoxville, TN, USA; c Anthropology, The University of Tennessee Knoxville College of Arts and Sciences, Knoxville, TN, USA

**Keywords:** Forensic sciences, forensic humanitarian action, forensic intervention, community entry Forensic anthropology

## Abstract

As forensic humanitarian and forensic human rights anthropology has continued to evolve, an ongoing concern in the field is meaningful engagement with survivors and the imperative to do no harm. For forensic anthropologists attempting to engage in grassroots forensic intervention, unaffiliated with an international investigation, means for effectively accessing and engaging communities has not been widely discussed. Here, forensic anthropologists draw on multiple, cross-cultural contexts to discuss methods and techniques for introducing forensic partnerships to communities. To do this, the scientist must consider their positionality as well as that of the stakeholders, develop effective local relationships, and consider a community-grounded approach. This paper argues that drawing on broader cultural anthropological training, ultimately informs one’s ability to gain entry into at-risk and vulnerable communities while minimizing harm. To illustrate this point, examples are drawn from Canada, Uganda, Cyprus, and Somaliland.

## Introduction

Forensic human rights and forensic humanitarian intervention in diverse scenarios (i.e. settler colonial, post-conflict, disaster, diaspora, and active conflict), continue to foster debate about the role and behaviour of scientists [1–3]. In recent years, forensic anthropology has increasingly emphasized the need for “defining stakeholders and establishing agreement on approaches to be taken”, and “recognizing local cultural factors” [4]. Forensic anthropologists assisting in the examination and repatriation of human remains are increasingly prioritizing collaboration with affected communities at the ground level [5,6].

Longstanding work of the Equipo Argentino de Antropología Forense (EAAF) and refugee diasporas as seen in the Mediterranean and US–Mexico border have brought attention to the complexity of working for and within surviving communities while navigating legal bureaucracies and local collaborations [7,8]. Contributions from international organizations, such as the International Commission on Missing Persons (ICMP) or the International Committee of the Red Cross (ICRC) scaffold much of the existing guidance, however, for forensic anthropologists affiliated with local or national organizations, the path for community entry is not always clear, especially when survivors may not be explicitly requesting forensic services.

Published forensic literature rarely discusses *how* one builds necessary relationships with community stakeholders prior to dialogues regarding the appropriateness of, or potential desire for, forensic interventions. To build those relationships, whether they are being actively sought or offered, several unanswered questions emerge, including: How might initial communication and collaboration with local communities be established? With what cultural beliefs, customs, concerns, or socio-political and socio-economic tensions do practitioners need to familiarize themselves? What are the most appropriate practical steps to build trust depending on such tensions?

Humanitarian forensic action (HFA) has garnered increasing support for the formation of interdisciplinary teams that respond to mass fatalities all over the world. The ICRC defines humanitarian action as activities that “seek to alleviate human suffering and protect the dignity of all victims of armed conflict and catastrophes, carried out in a neutral, impartial and independent manner, free of charge and framed under International Humanitarian Law” [1]. The use of forensic medicine and science within this humanitarian action framework is most often applied to post-conflict and disaster contexts where a desire exists to find, recover and identify the missing, thus helping to mitigate the trauma of the loss of life experienced by survivors. It should be noted, however, that in other post-conflict contexts collecting criminal evidence may be an additional goal or even the sole focus of forensic investigations, such as occurred with the International Criminal Tribunal for the former Yugoslavia (ICTY) or Rwanda (ICTR) which framed their involvement under International Human Rights Law. The goal of this article is not to emphasize one form of forensic involvement over another, but to explore how forensic practitioners may better engage with survivors to promote transparency and provide space for survivor engagement.

Forensic teams typically aim to assist in human remains recovery efforts in culturally sensitive ways, alongside training local staff in forensic anthropology methods. As such, forensic practitioners frequently seek to work in a reciprocal manner with communities by integrating the forensic work with local mortuary norms and cultural views rather than providing services in a top-down fashion. This work necessitates close collaboration with communities and survivors to ensure first that domestic stakeholders seek forensic intervention, rather than imposition of forensic measures as part of colonial paternalism. Further, next of kin considerations should guide the work with sustainable and holistic forensic practices built in-country going forward.

The authors argue that forensic community entry with survivors requires use of a socio-cultural lens to clearly position the scientist, develop sustainable local relationships, and frame community-based work. Hence, practitioners benefit from drawing on anthropological methods and training across the subdisciplines. Here, the authors discuss four ethnographically-informed forensic intervention scenarios to elucidate strategies for a practitioner seeking to build relationships with local communities for the purposes of forensic anthropological or archaeological work. In this text, diversity of cultural contexts also exemplifies potential barriers and alternative options for community entry. For the purposes of this inquiry, cases from Canada, Uganda, Somaliland, and Cyprus are discussed.

## Background information

Each of the chosen contexts delivers insight into politically and culturally diverse conflict-ridden societies with colonial histories; some have already engaged in mass identification agendas while others have not. While beyond the scope of this paper, arguably, diverse versions of transitional justice can be seen across these contexts as well. Transitional justice, meant to be a temporary alternative justice model to promote justice, reconciliation, and accountability, is now widely applied to scenarios of ongoing violence, oppression, and political tension.

### First Nations and Canada’s disappeared children of the Indian Residential Schools

As a settler colonial nation, the government of Canada (GOC) implemented over 150 Indian Residential Schools from which at least 6 000 children disappeared [9,10]. These institutions operated from the 1800s until 1996, and their rampant assimilation, physical abuse, sexual abuse, and medical experimentation on Indigenous children laid the groundwork for a 2006 class action lawsuit [11,12]. As part of the ensuing settlement agreement, the GOC outlined a transitional justice model—a framework in which forensic anthropologists frequently find themselves tasked with identifying, analysing, and repatriating human remains. Despite the presence of Christian cemeteries at each of the schools and the thousands of missing Indigenous children presumed to be interred, ultimately, transitional justice coordinators did not implement repatriation efforts. Investigations into human remains at residential facilities are not unusual and have occurred in contexts such as Ireland’s Mother and Baby Homes [13], US Florida Industrial School for Boys [14], or the US’ Carlisle Indian Industrial School [15].

Disputes between Indigenous communities and government officials continue to this day regarding the treatment of Aboriginal remains considered historical, regardless of clear ties to living peoples and ongoing violence. Transitional justice efforts failed to address the dearth of forensic options available, sensitization opportunities, and the increasing demand for repatriation. In economically-similar countries, like the US, repatriation of Indian Boarding School children’s bodies has proven meaningful to Indigenous communities [16].

While the GOC elected to avoid engaging in forensic identification, fieldwork experiences regarding rapport-building and community entry during active phases of transitional justice can inform future forensic humanitarian and human rights interventions.

### Uganda’s mass, unmarked, and “improper” graves

Like Canada, Uganda has a colonial and extractive history—one which has contributed to devastating levels of internal conflict, with the most recent being the war between a rebel, guerrilla group, the Lord’s Resistance Army (LRA), and the government of Uganda (GOU) that resulted in countless mass killings under the leadership of President Yoweri Museveni [17–19].

No independent agency has conducted a conclusive investigation into the number of missing [18,20–23]. Estimates from the 1986—2006 conflict range from tens of thousands to hundreds of thousands of deaths and missing [24,25]. Decades of war and internal displacement created a geography of mass, unmarked, and otherwise problematic graves [26,27]. For the last 10 years, a team of anthropologists has been working with rural community members of the most impacted of Uganda’s over 50 ethnic groups—the Acholi—Ugandan government officials, and non-governmental organizations (NGOs) to assess the desire, feasibility, and implementation of forensic capacity building and intervention [28]. Cultural hierarchies and norms specific to the Acholi, Ugandan local and national governmental structures, and NGO and civil society interlocutors require careful navigation and negotiation at the start, providing insight into how independent forensic consultants or “ground up” initiatives may be established.

#### Somaliland’s extrajudicial killings under Siad Barre

Somalia, including the self-proclaimed autonomous region Somaliland, has seen several cycles of violence in the aftermath of colonialism, some of the most volatile under dictator Mohamed Siad Barre. After seizing power through a coup in 1969, Siad Barre unleashed a totalitarian regime that was especially atrocious in present-day Somaliland [29]. Somaliland self-declared autonomy from Somalia after Barre’s rule ended in 1990, and the region began investigating war crimes committed under the former dictator. The exact number of people killed under Barre is not known; estimates for Somaliland ranged from 5 000 to 50 000 in the 1980s, though local officials have placed the number closer to 100 000 or even 200 000 [30].

Tens of thousands of people were killed in 1988 alone as the government air bombed Hargeisa, the Somaliland capital, forcing approximately one million people to flee to the countryside or neighbouring Ethiopia [31,32].

The Somaliland War Crimes Investigation Commission (WCIC) was established by the Somaliland government in 1997 and has since led investigations into the Siad Barre atrocities. The WCIC has been supported by the Equipo Peruano de Antropología Forense (EPAF), or the Peruvian Forensic Anthropology Team, since 2012. The work has included mass grave exhumations, skeletal analysis, and reburial of dozens of victims of the Siad Barre regime.

During the investigation, EPAF has conducted the human remains recovery operations while relying on the WCIC for community outreach. However, there are hundreds of mass graves in the region and few families know where their relatives are buried. In addition, many individuals are still missing and several survivors cannot confirm whether or not their missing family members were killed during the Siad Barre regime, complicating outreach efforts. Though some family members have participated in the forensic investigations and reburial ceremonies, the vast majority of survivors have not taken part nor are they aware whether their family members have been exhumed and reburied.

Ethnographic research conducted in 2019 sought to investigate family perceptions of the investigations as well as how family members would prefer for future forensic practices to unfold. As such, both family members who had participated in the forensic work and those who had little awareness of the investigations were engaged. At the onset, this work necessitated dialogue between the researcher, assistants, and survivors regarding forensic conduct to shine light on the history and purpose of the investigations. Additionally, trust and rapport were maintained through collaborative discussions around local mortuary customs and survivor experiences, demonstrating how community entry can hinge on the researcher’s positioning while further trust-building can be established through reciprocal discussions.

### Greek- Cypriot and Turkish- Cypriot disappearances

“The Cyprus problem” derives its name from the 1972 conflict that pitted

Greek-Cypriots and Turkish-Cypriots against each other and resulted in the longest United Nations (UN) peacekeeping mission to date [33]. To this day, UN soldiers patrol a narrow strip of no-man’s-land separating the two sides from one another. Similar to Uganda, as Cypriots gained independence from the British Empire after World War II, political and ethnic rivalries spurred divisions between the two major ethnic groups as they attempted to fill the power vacuum left by the vacating empire. Tensions flared in the 1960s with inter-ethnic killings and disappearances. Domestic and international efforts, while at first successful at reducing conflict, ultimately failed when a faction of Greek-Cypriots, fixated on uniting Cyprus with mainland Greece, attempted a military coup of the government. The conflict drew both Greek and Turkish forces into the engagement resulting in further killings and disappearances of both military personnel and civilians. Turkish-Cypriot residents were expelled from the southern portion of the island; likewise, Greek-Cypriots were forced to leave the north.

From the initial aggressions in the 1960s and the later 1972 war, hundreds of people disappeared. The disappearance of family members has caused extensive consternation on both sides, where demands for government action spurred the 1980 formation of the Committee on Missing Persons (CMP) in Cyprus [34]. The CMP has sought to resolve the fate of the missing from the 1960s and 1972 conflicts. However, one challenge of “the Cyprus Problem’’ has been the inability for the two sides to agree on problem-solving measures, causing the CMP to languish for years in all but name only. The CMP solved nothing until 2005 when it became active in the search and identification of the missing.

In these four diverse contexts and regions of the world, the authors examine how researchers and scientists engage communities and local actors, arguing that a socio-cultural lens in the scientist–survivor relationship is critical for accessing communities and engaging stakeholders. First, the positionality of survivors and scientists illustrates how perceptions will influence community entry. Following this, developing local relationships as a means of engaging potential stakeholders is discussed. Finally, the authors consider how a community-based approach can ground survivor-centred interventions and facilitate dialogue with stakeholders.

## Contextualizing the positionality of survivors and the scientist

Part of front-end forensic intervention work entails research into the cultural context for which one intends to provide services. While ethical concerns, respect for diverse cosmological viewpoints, and evidentiary reasons drive this foundational step, there is also the matter of community perception of the investigators. Regardless of geography or if assistance is sought or offered, it is common that at-risk or vulnerable populations are sceptical of “outsiders” [35,36], especially if there is a belief that the scientist could be affiliated with a corrupt government. A question to ask oneself is how the positionality of the scientist may be perceived? One can safely assume that arriving with consent forms, surveys, or powerpoints in hand at the time of entry does not facilitate building the necessary rapport to gain community entry [37]. Rather, there will likely be a series of conversations, meetings, and communications to start building the investigator–survivor relationship. While forensic experts often claim the position of impartial scientists, survivors do not necessarily perceive scientists in this way as evidence and science can be manipulated or misrepresented to serve political agendas. That being said, using the contexts described previously, modes of positioning the scientist are used to exemplify challenges and strategies that may be encountered.

During Canada’s implementation of their national transitional justice framework, the author Kim engaged Indigenous populations regarding the topic of the missing children from the Indian Residential School System. Given Canada’s settler colonial status, Kim would not have gained community access without being able to demonstrate independent research status — unaffiliated with governmental agendas or truth commission reporting. Even as an independent consultant, affiliation with a foreign academic institution alone did not dissuade uncertainty regarding Canadian government involvement, and in fact, the suspicion initially deterred some from interacting. However, understanding of socio-cultural norms of Eastern Woodland Indigenous groups facilitated networking amongst Indigenous Canadians, despite tribal or band differences. Familiarity with both past and current forms of colonial and political violence, pressing local Indigenous human rights issues, and Indigenous cultural practices were integral in rapport building and community entry. At the same time, the independent status of the forensic scientist and known Indigenous affiliations hindered access to government-funded inquiries into the missing children. Some national entities viewed the position of the independent forensic interlocutor as adversarial once Indigenous community affiliations were established, and professionals engaged in the official missing children inquiry were advised not to communicate with the author. While beyond the scope of this paper, it does bring to light the need to also build relationships with law enforcement, governmental, or investigative entities who are stakeholders in an investigation.

Moreover, one should be aware that for survivors, distinguishing between legitimate and illegitimate forensic intervention can be challenging. For example, in Canada, a man claiming to be a forensic investigator was spreading misinformation about Indigenous remains allegedly buried at the residential schools [9]. This created precarity in identifying oneself as a forensic investigator, as some had developed false beliefs about forensic investigation based on the man’s actions; others knew it was a charade but remained sceptical because other, legitimate, forensic investigators did not intervene or were so disengaged from the Indigenous experience that they were unaware. These issues underscore the idea that positionality, or rather perceived socio-political standing of the investigator impacts community entry— which can be overcome in part with meaningful knowledge of the cultural context.

In the case of Uganda, the authors and their colleagues aimed to engage survivors to ask what they desired be done with mass, unmarked, or otherwise problematic graves and to provide capacity-building. In this context, current President Yoweri Museveni remains in power after governing during the war with the LRA. A number of mass graves and civilian deaths have been attributed to the National Resistance Army and its successor, the Uganda People’s Defence Force, complicating the potential for forensic intervention [20,23,27].

Additionally, non-governmental agencies and foreign aid programmes have proliferated throughout Uganda [38], contributing to confusion regarding types and purposes of intervention [39]. These factors led to continued perceptions of the forensic research and capacity-building team as either an NGO, there to provide tangible supplies, or worse, agents of a corrupt government gathering information on those who may speak against it [40]. These misconceptions, in part due to the team’s status as American interlocutors, created a need to clarify the team’s positionality, not only at the start, but frequently and often throughout the process.

As part of contextualizing the positionality of the scientists, the team engaged with hundreds of community members. Even though villages may seem distant from one another, the communication networks performed quickly and efficiently. It became apparent that survivors talked amongst themselves to verify the consistency in what the scientists were stating. Dierickx et al. [41] report a similar phenomenon in a sensitization and recruitment model for a medical trial in sub-Saharan Africa. Community sensitization regarding malaria and the subsequent study had a domino effect as “the first-hand, accurate information delivered at the community meetings was cascaded as ‘lay’ information to those other community members who could not attend the sessions”. These informal information networks were key in establishing realistic expectations and building rapport and reliability between community members and investigators. In the Ugandan case, transparency efforts were conscious and intentional. For instance, the team reiterated realistic expectations of their work, including the possibilities and limitations associated with forensic science. These conversations were sometimes arduous and at times disheartening for some rural Ugandans as it was revealed that DNA identification relied on comparison of human remains to reference samples from living relatives — no machine exists that can produce an identification from only the unidentified sample.

In rural areas of Uganda, there may have been DNA knowledge in terms of paternity testing or examination of remains in terms of autopsy, but little knowledge of excavation, analysis, and repatriation processes. A forensic investigation in Lukodi, presumably orchestrated by the ICC was mentioned by survivors as a point of reference, and in this instance “forensic doctors” reportedly had survivors exhume decomposing remains of loved ones for analysis with little explanation given. Other non-governmental organizations facilitated or supported exhumations or burial rites under a wide umbrella of reasons, ranging from necessary removal of remains obstructing development to reconciliation efforts [42]. Contextualizing and positioning forensic interventions from non-forensic exhumations was an important part of gaining community entry.

In Somaliland, the entry into survivor communities hinged on the researcher’s dual position as a forensic insider and an independent investigator. There were similar concerns as those in the Ugandan and Canadian contexts, including the need to communicate and differentiate legitimate forensic practices from rumours or layperson conduct. Here, however, forensic investigation had been ongoing for 7 years, so engaging communities encountered unique challenges in comparison [30]. The author Elgerud had participated in the forensic recovery work in 2014 and subsequently conducted independent ethnographic fieldwork in 2019. The latter aimed to assess local familiarity with the human remains recovery operations and gauge family views of the work in order to address the impact of the forensic investigation while identifying preferred future trajectories.

During fieldwork, Somaliland families were rarely apprehensive about participating in interviews conducted by a foreign researcher, perhaps due in part to Elgerud’s position as an independent ethnographic researcher as well as a former forensic interlocutor in the local investigations. Additionally, Elgerud’s identity — a Swedish citizen affiliated with an American university — facilitated initial rapport building as several informants had close family in Sweden or the US, prompting conversations about diaspora family members or personal experiences abroad. Due to displacement during the Siad Barre regime, many families in Somaliland saw relatives relocate to North America, Europe, and the Middle East, and most retain close connection with the diaspora communities [43]. In contrast to the authors’ experiences amongst Ugandan survivors, being an outsider proved facilitative in the Somaliland context, further conveying the importance of familiarization with local socio-politics and history for community entry.

Elgerud’s dual position allowed for navigating tensions associated with the investigation while clearing up misconceptions about forensic procedures. A reoccurring obstacle to the investigations and a point of contention among family groups in Somaliland, was the lack of knowledge about the aim and scope of the investigation. Because of this, some family groups had even acted hostile toward the international investigators, accusing them of stealing the bones to make plates and cups that they could sell in their home countries. During the ethnographic work, several community members expressed concern over exhumations, stating the Islamic prohibition on opening a grave for any reason or for any other than “legitimate” reasons; the “intention” behind exhumation was often cited by informants who were concerned about religious restrictions. Such concerns typically led to lengthy discussions about the purpose of forensic investigations, where the researcher was able to provide insight into forensic practices and family members defined legitimate reasons or intentions for conducting such practices in different contexts. These reciprocal discussions would further facilitate researcher–informant rapport, again highlighting the importance of the perceived positionality of the forensic investigator as well as open dialogue for the purpose of building and maintaining trust.

Trust building itself also allowed for further community entry in the Somaliland case as many family members would allow the researcher access to part of their clan network after trust had been built between the researcher and the survivors. The clan system of Somali societies interconnects the population, which assisted in “snowball sampling”; each research participant was able to connect the researcher with several additional family members of those who died during the atrocities once a reciprocal relationship had been established. Elgerud’s experiences in Somaliland demonstrates how understanding on-the-ground context and stakeholder positions—in this case informed by the political landscape and kinship structures—can help the scientist identify and navigate potential barriers or opportunities.

In the case of Cyprus, forensic community entry was demanded by concerned family members who ultimately forced the two sides to begin forensic investigations—generating a ground-up call for scientific input. The Greek-Cypriot government insisted the missing were alive, regardless of contradicting evidence, and demanded their return. In contrast, the Turkish-Cypriot government acknowledged their missing were dead, elevating them to Martyrs who sacrificed for the good of their people, honouring them with monuments and in speech, and thus rejecting the need to recover them [44]. Many surviving family members of Martyrs were grieving, and while the accolades helped mitigate some of the pain, for some there was an underlying frustration at government inaction to find those who died [45]. Eventually, two Greek-Cypriot civilians snuck into a cemetery at night and attempted to exhume the graves they believed were their husbands’. They were caught and their story exposed the inaccuracy in the government narrative that claimed full support of family members.

The incident opened a national dialog regarding the missing and ultimately resulted in the Greek-Cypriot government beginning unilateral forensic investigations, with the assistance of Physicians for Human Rights (PHR), on that side of the border. While the Turkish-Cypriot government was upset at this unilateral action, it forced discussion on their side of the border and culminated in CMP becoming active in a joint search and identification of missing persons.

Initially, the CMP contracted with the Argentine Forensic Anthropology Team (EAAF) to help train and guide their new Cypriot staff in large-scale identification projects. The author Tuller was invited by the EAAF in the fall of 2006 to assist in this effort and primarily helped guide the archaeological teams—experience which proved useful when returning to Cyprus in 2018 to conduct research regarding survivor and practitioner perspectives on the identification process. Tuller had maintained contact with a number of forensic staff over the years, monitoring their progress and was still known by some of the CMP leadership.

Family members on both sides of the border have been interested in the progress CMP has made and have engaged favourably with the CMP forensic staff. The staff strives to make their operations transparent and counter false rumours and narratives of their work. Not only does CMP release regular progress reports, it also holds regular family updates, conducts tours of their laboratory for family groups and community representatives, and participates in public speaking engagements highlighting their work, further building the trust between the forensic operations and the communities. While not everyone is satisfied with the progress or cooperative nature of the CMP, the organization and its forensic staff receive the support of most of the family organizations and communities on both sides. Local staff, familiar with the historic and cultural contexts, can build these bridges if they engage their communities in open and honest ways. Forensic outsiders should be aware of these links and utilize their colleagues or staff’s insights into how best to engage the population.

Tuller was able to parlay his past work experience at CMP and relationships with its forensic staff into support for his research. After gaining permission to engage its staff from CMP, his colleagues helped with introductions into their two communities. The relationship the CMP forensic staff had developed over the years, positioning themselves as transparent and cooperative with their communities, was instrumental in this case. Tuller’s initial introductions also acted to assuage any misgivings participants had of the research or the positionality of the foreign researcher. The key here was the established trust developed and nurtured over time between the families and the CMP forensic practitioners from those communities. Tuller himself did not have any specific contacts with surviving family members outside of the CMP staff.

Across contexts, the perceived perception of the forensic scientist impacted community engagement, but in diverse ways, dependent on socio-political climates, histories of forensic presence or absence regarding remains, and cultural norms. From this, we see that while each scenario is different, general factors can be taken into consideration when preparing to engage with communities. One need to ask the relationship between current governments and survivors, pressure points for ongoing colonial relationships, historical and current interethnic disputes, layperson perceptions of any real or believed forensic interventions, or even the presence of NGOs and other “outsiders”. Regardless of contexts discussed here, transparency, communication of realistic limitations, and deep understanding of the socio-cultural and political context has consistently assisted in positioning the forensic scientist to engage with the community positively. However, these are just the first steps in gaining community entry with survivors. As each context has shown, developing local relationships proves integral and can be accomplished through alternative routes.

## Developing local relationships

Forensic scientists, and anthropologists in particular, have been critiqued for entering communities with little preamble and conducting work restricted by governmental demands, international prerogatives, and academic ideologies [7,46,47]. As part of collaborative forensic humanitarian action or human rights intervention, the depth of community entry and acceptance of the scientist relies on developing local relationships and understanding the stakeholder landscape, socially or politically contentious issues, and pinch points that may develop. While the scientist establishes their positionality, Tuller’s experience in Cyprus prompts the question, with whom does one seek entry? What relationships assist in developing the social capital with the affected community? To legitimise one’s presence and begin building scientist–survivor relationships, introducing oneself to reputable, local community organizations can facilitate the process. However, again, one must have done enough prior inquiry to identify with which organization not to align.

In the case of British Columbia, Canada, engaging with local organizations dedicated to Indigenous visions facilitated building relationships in the urban, multi-ethnic Indigenous community. For instance, while one major non-profit organization, the Indian Residential School Survivor Society (IRSSS) was an obvious organization to contact, extensive collaboration with organizations such as the Indigenous religious organizations, Indigenous media outlets, and local community organizers allowed for development of deep-seated scientist–survivor relationships. Indigenous survivors also organized around demonstrations, protests, tourism, and cultural events, allowing Kim to identify tensions between regional or national government and Indigenous peoples while networking and identifying stakeholders with a vested interest in forensic investigation. There were also relationships in which fostering or developing ties could create barriers to community entry. Several prominent Indigenous officials, for example, were perceived to be corrupt. Aligning oneself with them could create barriers for the scientist as significant swaths of the community would either avoid interaction or feign superficial interaction with associates of those who were untrustworthy.

Similar to Elgerud’s experience with clan affiliations, and Tuller’s referrals by the CMP, snowball sampling assisted in forming local relationships. Being a relatively tight-knit community, engaging with intertribal organizations allowed contact and communication with survivors from diverse ethnic groups, such as the Haida, Tlingit, Tsleil Waututh, Musqueaum, Kwakwaka’wakw, and Squamish. Moreover, in this context, positionality as a political outsider sometimes mollified suspicions that could arise from developing affiliations with individuals whose beliefs or actions could be perceived as controversial. Openness to understanding the nuances of Indigenous–Settler and Indigenous–Indigenous politics assisted in making headway with relationship-building and reinforced the positionality of the investigator.

There may be culturally-specific barriers to identify in the pursuit of local relationships and networks. For instance, in the case of Uganda, two sets of leadership needed to grant approval for forensic sensitization. The first being local government officials who must approve formalised paperwork; the second being cultural leaders, *rwot*, or clan chiefs. While identifying local government officials may be anticipated, the approval process, meetings, explanations of work, and scheduling can take months, particularly if working in more than one district and with limited resources. The clan chiefs, in this case, have their own hierarchical structure and cultural norms that needed to be followed during the establishment of the collaboration process.

Awareness of culturally specific gatekeepers can inform development of necessary relationships for community entry.

In addition to clan chiefs and local government, investigators in Uganda were able to identify several NGOs that worked closely with survivors documenting witness statements regarding war-related violence. Because documentation naturally bridged the NGO interests and investigator interests, collaboration with NGOs facilitated community access. The research team was able to reach out to communities that were familiar with the NGOs and had pre-existing rapport. It should also be noted that during Ugandan work with communities, local individuals collaborated with the research team to assist as translators and peer review transcriptions, amongst other tasks. Several of the collaborators who served as translators were relatively well known—one through the music industry, another due to family governmental ties, and yet another due to NGO work. They provided a known presence and association for the investigators. Perhaps more integral than the translation itself, they also assisted in explaining points of potential cross-cultural confusion between the Acholi participants and American investigators or issues rife with political tensions.

This also proved true for the Somaliland fieldwork, where close relationships with local assistants and translators assisted in communicating concerns that may otherwise have been lost in translation and hindered development of local relationships. For instance, Somali society is largely organized around Islamic practices, and several cultural, legal, and political concerns are rooted in religious principles. Local translators were imperative for this reason, since they were able to more thoroughly convey informant concerns that were grounded in Islamic theology and conduct, of which the researcher only had surface-level knowledge but needed to grasp for both relationship building and data interpretation. Thus, collaboration between the researcher and informants was made possible by assistants acting as translators as well as discussants and mediators.

Community entry in Somaliland also entailed liaising with local organizations, primarily the governmental WCIC, which have been leading the forensic investigation and collaborating with EPAF. However, the WCIC has limited connections with family members since only a minority of survivors from the Siad Barre regime atrocities know where their loved ones are buried. Thus, while the WCIC supported Elgerud during fieldwork, affiliation with the organization was not imperative for connecting the researcher to widespread survivor communities. The researcher’s relationship with the WCIC still proved important to facilitate the ethnographic work, as some informants hesitated to participate in research that was not supported by the Somaliland government. Elgerud had collaborated with the WCIC as part of the EPAF investigations in 2014, and later revived the connection with the organization while conducting fieldwork in 2019. As such, tensions surrounding the research were able to be overcome as those who were closely aligned with the WCIC could be assured of the researcher’s amicable relationship with the organization. At the same time, it was important to be clear that the project was independent from WCIC, as some sceptical informants did not want to be associated with a governmental institution. This transparent relationship with WCIC, alongside informant’s confidentiality under the research protocol, provided a safe and trusting environment for participants and the researcher. In similarity to the Canadian context, navigating political tensions and building rapport with local organizations were imperative for comprehensive, open, and safe research in Somaliland.

The importance of careful consideration regarding which organizations to approach can also be seen in Cyprus. There, the CMP is a bi-communal organization physically located in the no-man’s land between the two formerly warring sides. The dynamics of who speaks to which side becomes important. While the CMP bi-communal cooperation has been praised by some as a step towards solving the “Cyprus Problem”, CMP understands that this cooperation may not be viewed as progress by all, especially family members who could harbour ill feelings towards people on the “other side”. When engaging with Greek-Cypriot community members, the CMP will offer Greek-Cypriot forensic staff to speak, and Turkish-Cypriot forensic experts for the Turkish-Cypriot community. In this manner, families and community members have someone from their own community to speak with in their native language, helping them to understand the complexities of forensic investigations, and providing a more culturally recognizable and comfortable setting to ask direct questions. Having someone from a different community attempt to engage on such sensitive subjects like the death of a family member could bring unintended results to the relationship. As it was family members that forced their governments to engage in a large-scale forensic identification project, the CMP wisely hired local forensic practitioners and regularly uses them to directly explain their operations to the communities they are from.

In all contexts, collaboration with local entities that share identity with, and are often comprised of survivors has proven integral to forensic capacity building and communication. Bridges like this to the local population have been found to be effective in community relations in non-forensic contexts as well, modelling approaches that can be applied, interrogated, and modified for the international forensic community. These observations align with Eisenman and colleagues [48] who found that hiring local health care workers who were bilingual to lead disaster preparedness sensitization for immigrant communities in the US led to increased self-reported preparedness. Use of local language, development of local networks, and thoughtful collaboration with local entities or individuals can assist in gaining community entry and developing rapport in forensic contexts. However, collaboration should be grounded in mutual interests and agency, lest it be paternalistic and prescriptive.

## Participatory action or community- based forensic work

One way to temper prescriptive approaches would be the utilization of collaborative models such as participatory action or community-based work [49–51]. As professionals build local collaborations for forensic human rights anthropology community entry and interventions, community-based participatory research (CBPR) and similar frameworks can undergird the work. Broadly speaking, CBPR relies on collaborations between the researcher and target population throughout the duration of the project, including development of the research questions; instead of participants, affected communities become collaborators. This perspective is beneficial in contexts in which survivors are seeking assistance as well as when sensitization is presented as an option. Translating this into community entry includes providing introduction to forensic concepts and capabilities and first identifying 1) if communities are interested in forensic intervention, 2) if they would like more information before deciding, and 3) what their priorities and challenges are regarding human remains. Developing sensitivity to cultural issues (e.g. political, economic, spiritual) that may inform their needs must be part of the dialoguing process. In part, the goal of frameworks like CBPR are to contribute to resolving issues identified by the target population, rather than those of the researcher/practitioner. This approach facilitates community entry as the survivors recognize that researchers are there to assist in, rather than control, the process.

In lieu of mandating the forensic actions that must be taken, a community-based approach builds a relationship between the scientists and the survivors. For vulnerable or marginalized populations that have historically been studied or controlled by dominant groups, clarifying the desire to assist, rather than direct, is especially imperative. Research in Canada, a nation-state with a history of continued settler colonialism exemplifies the need for a community-based approach. In an attempt to address the residential school history and missing children, members of the Assembly of First Nations and survivors pursued a transitional justice model that looked unlike the final, negotiated approach found in the resulting settlement agreement. A community-based approach emphasizes a view shared by diverse Indigenous groups that understood the missing children and residential schools to be a part of a continuum of violence, rather than an isolated point in history. Indigenous children displaced *via* the child welfare system, the disproportionate number of missing and murdered Indigenous women, and continued desecration of Indigenous graves all intertwine with the investigation into the residential school disappeared. This, combined with reserves, homelessness, urban Indigenous life, and the reality of interethnic heritages, complicate the logistics and practicality of a repatriation process.

Understanding community goals or (un)desire for forensic intervention will inform if such a process should take place, and if so, to what end and with what methods.

In the case of Uganda, another country with a history of colonization, relationships were built between the investigators and local leadership, NGOs, civil societies, and individuals to ascertain what they felt forensic anthropology could provide, to develop an understanding of socio-cultural challenges that human remains presented to local communities, and to understand how and why forensic intervention may *not* be desired. For example, graves of improperly buried remains can sometimes serve as visual markers of devastation in Acholiland, and therefore provide incentive for government financial restitution to that community. Forensic exhumation and repatriation were perceived by some negatively as a way to erase the mass atrocities from the landscape when they sought to preserve it.

However, in some instances, communities requested site mapping of graves, for a variety of reasons. For some, this would assuage fears of burials becoming lost to failing memories [52]. The site reports also assisted in conveying forensic approaches to a scene by showing how the graves were documented prior to exhumation and what type of information formed part of the forensic report. The community-based research included the use of images to convey information about forensic practices that were not well known in some of the local communities. Both the site mapping and the use of images helped rectify misconceptions about forensic investigations and encouraged engagement between community members and investigators. Participants responded to the community-based approach which facilitated relationship-building and community entry.

Similarly, during fieldwork in Somaliland, the researcher sought to gauge local family and survivor views on forensic investigations. Here, the forensic topics were assuaged by an emphasis on Islamic mortuary practices, which helped shift the power dynamic away from the investigator. This approach was found to support trust-building and community collaboration by pivoting the discussion toward local customs and everyday concerns rather than the often less familiar forensic work. The amalgamation of forensic topics and Islamic mortuary practices was informed by Al-Dawoody’s [53] research into preferred treatment of the dead amongst Islamic communities during ICRC HFA interventions. Al-Dawoody is an Islamic scholar who has worked with the ICRC for several years. During this time, he has found that there are community needs related to Islamic approaches to death and burial that can be accommodated during the human remains recovery and analysis operations. For example, the use of refrigerated units can help mitigate the traditional need for burial within a short time span found in most Islamic communities. In drawn-out disaster and conflict recovery settings, refrigerated units for storing human remains would save families from the hurtful odour of decay and encourage burial at a later time after bodies have been identified [53]. The act of handling human remains after disasters and conflicts is another area of concern. It is often preferred for Muslim practitioners of the same sex as the dead to handle and wash the dead. However, if this is not possible, such as in mass atrocity or mass disaster situations, other practitioners can perform the same services based on “necessity”, which can override religious prohibitions [53]. These and similar topics related to Islamic burial practices were foundational for collaboration during the Somaliland research and provide insight into how forensic work can best be performed to respond to family concerns.

Focusing on the overlap between forensic work and local mortuary practices thus facilitated a community-driven approach to the investigations while shifting power dynamics in favour of the affected communities.

Warfare, across contexts, is ultimately political, and the narratives derived in its aftermath are formed to support specific governmental goals. The same can be seen in the case of Cyprus, where the political narratives of the two governments failed to conform to family needs over time. While the exhumation in the Greek-Cypriot cemetery by widows searching for their husbands may have failed, it did start the conversation as to what can be done with burials of unknown people from the war, and what could be done with possible clandestine burials.

International forensic experts were introduced to share their thoughts and knowledge, to include Tuller, and as the CMP transformed from just another forum of debate between the Greek and Turkish sides to a bi-communal forensic identification operation, both family and government officials began to learn that large-scale identification processes were possible. More importantly, the conversation between forensic experts and these communities provided a platform for the families to express their desires and for the governments to understand how a forensic intervention could assist. Based on the understanding of family desires and forensic capability, the governments shifted their narratives to give room for the CMP bi-communal forensic investigation and identification project.

## Concluding remarks

Earning entry into communities of human rights survivors as a non-domestic forensic investigator requires an understanding of the socio-cultural contexts in which one works. The four historically and culturally diverse contexts of Canada, Uganda, Somaliland, and Cyprus evidence strategies for building rapport and engaging survivors. Such community access can be facilitated by transparency on the part of the forensic scientist vis-a-vis clear communication of realistic and unrealistic goals and understanding how political dynamics fuel or affect suspicion of foreign investigators. To achieve this, timelines for intervention do not need to be protracted, as rapid assessments and other methodologies can be utilized. Rather, we advocate for interrogating existing notions of “collaboration” or “engagement”, through employing a socio-cultural lens. Developing local relationships *via* existing social infrastructure also facilitates community entry as trusted partners can vouch for the integrity of the researcher. This may require networking through several degrees of separation or leveraging social capital until reaching the target audience. Finally, making use of a community-based or participatory approach empowers survivors and mitigates power hierarchies that may inhibit stakeholder buy-in. This reciprocal relationship, including the information regarding forensic practices, also encourages participants to refer their close and distant relatives, enhancing the reputability of the investigator.

At the same time, understanding of the local socio-political context reveals obstacles to entry, such as the persisting belief amongst Ugandans that the forensic interlocutors would behave as an NGO. Power dynamics between forensic investigator and survivor will be entangled in these political contexts, as interlocutors could be viewed as channels for resources, colonial collaborators, or wielders of knowledge or authority. Similarly, survivors understand that forensic evidence can be framed, reframed, or manipulated to serve diverse governmental and non-governmental agendas, derailing the notions of scientific neutrality in which many investigators believe.

The complexities of the scientist–survivor relationship merit deeper discussion as forensic humanitarian and human rights anthropology continues to evolve, increasingly emphasizing the needs of stakeholders and historical and political contexts. To accomplish goals such as these, success hinges on survivor involvement in not only the forensic intervention, but the events leading up to and determining the type of intervention as well. Here, this piece builds on critiques and contributes insight into *how* survivors can be engaged meaningfully by forensic scientists. The discussion here stimulates further dialogue on the practical realities of implementing forensic humanitarian action for both non-domestic practitioners and local stakeholders, the logistics of which are frequently absent from academic publishing. Through examining *how* community entry with survivors can be accomplished, survivor involvement and priorities can be maximized on the part of the forensic interlocutor.
